# Peptide Receptor Radionuclide Therapy Using ^90^Y- and ^177^Lu-DOTATATE Modulating Atherosclerotic Plaque Inflammation: Longitudinal Monitoring by ^68^Ga-DOTATATE Positron Emissions Tomography/Computer Tomography

**DOI:** 10.3390/diagnostics14222486

**Published:** 2024-11-07

**Authors:** German Rubinstein, Harun Ilhan, Peter Bartenstein, Sebastian Lehner, Marcus Hacker, Andrei Todica, Mathias Johannes Zacherl, Maximilian Fischer

**Affiliations:** 1 Department of Nuclear Medicine, LMU University Hospital, LMU Munich, 81377 Munich, Germanymathias.zacherl@med.uni-muenchen.de (M.J.Z.); 2 Department of Medicine IV, LMU University Hospital, LMU Munich, 80336 Munich, Germany; 3 DIE RADIOLOGIE, 80331 Munich, Germany; 4 Division of Nuclear Medicine, Medical University of Vienna, 1090 Vienna, Austria; 5 Medizinische Klinik und Poliklinik I, Klinikum der Universität München, Ludwig-Maximilians-Universität, Marchioninistrasse 15, 81377 Munich, Germany; 6 DZHK (German Centre for Cardiovascular Research), Partner Site Munich Heart Alliance, 80802 Munich, Germany

**Keywords:** atherosclerosis, plaque imaging, DOTATATE, PET/CT, inflammation

## Abstract

**Background:** Atherosclerosis and its sequels, such as coronary artery disease and cerebrovascular stroke, still represent global health burdens. The pathogenesis of atherosclerosis consists of growing calcified plaques in the arterial wall and is accompanied by inflammatory processes, which are not entirely understood. This study aims to evaluate the effect of peptide receptor radionuclide therapy (PRRT) using ^90^Y- and ^177^Lu-DOTATATE on atherosclerotic plaque inflammation. **Methods**: Atherosclerotic plaques in 57 cancer patients receiving PRRT using ^90^Y- and ^177^Lu-DOTATATE were longitudinally monitored by ^68^Ga-DOTATATE PET/CT. The target-to-background ratio (TBR) and overall vessel uptake (OVU) were measured in eight distinct arterial regions (ascending aorta, aortic arch, descending aorta, abdominal aorta, both iliac arteries, and both carotid arteries) to monitor calcified plaques. **Results:** PET/CT analysis shows a positive correlation between calcified plaque scores and the ^68^Ga-DOTATATE overall vessel uptake (OVU) in cancer patients. After PRRT, an initially high OVU was observed to decrease in the therapy group compared to the control group. An excellent correlation could be shown for each target-to-background ratio (TBR) to the OVU, especially the ascending aorta. **Conclusions:** The ascending aorta could present a future reference for estimating generalized atherosclerotic inflammatory processes. PRRT might represent a therapeutic approach to modulating atherosclerotic plaques.

## 1. Introduction

Atherosclerosis and its sequels, including aorta disease, coronary artery disease, and peripheral artery disease, are still a significant burden in western society [1].

In atherosclerosis, there is an interplay of inflammation, necrosis, fibrosis, and calcification [2]. Recently, inflammation has been spotlighted as a dominant player in atherosclerosis progression and, therefore, the clinical manifestation of the otherwise silent disease [3]. Risk factors such as hypercholesterinemia hypertension, tobacco use, and diabetes contribute to the progression of atherosclerotic plaques [3].

The search for the best modality to predict which atherosclerotic lesions are prone to rupture is still ongoing. Historically, angiography, computer tomography (CT), and magnetic resonance imaging (MRI) are based on the basic morphological aspects, providing information about the degree of stenosis and, therefore, the hemodynamic relevance of atherosclerotic plaques. However, these imaging technologies cannot estimate the inflammatory activity or the vulnerability of atherosclerotic plaques. The fusion of morphological aspects paired with positron emission tomography could help overcome these limitations and, therefore, offers tremendous potential in diagnosing and treating atherosclerosis [2,4].

Previous studies depict 2-deoxy-2-[fluorine-18]fluoro-D-glucose (^18^F-FDG) in imaging atherosclerosis. ^18^F-FDG is a useful tool in giant cell arteritis [5] and illustrates the activity of immune cells (e.g., macrophages) in atherosclerotic plaques in the carotid arteries of symptomatic patients [6]. Previous correlations illustrate ^18^F-FDG uptake in carotid plaques and the macrophages in histology and, therefore, noninvasively measure inflammation in patients [7]. Furthermore, ^18^F-FDG uptake in the left anterior descending artery (LAD) is associated with cardiovascular risk factors and plaque burden in patients [8]. ^18^F-FDG uptake also predicted future vascular events when quantified in atherosclerotic lesions [9] and detected the anti-atherosclerotic effect of statins [10]. Besides the available literature showing the benefits of imaging atherosclerotic lesions, ^18^F-FDG bears several limitations. The uptake of ^18^F-FDG is unselectively into all active metabolic cells. The uptake into vessels is influenced by plaque hypoxia and different microcirculatory conditions (reviewed in [11]). Focusing on the inflammation of cardiac arteries, the proximity to the heart muscle is still an obstacle limiting accurate analyses [12], especially in diabetic patients [13]. In recent years, the knowledge of immune cells, especially macrophages, and their pivotal role in cardiovascular disease and inflammation grew immensely [14].

Macrophages express the somatostatin surface receptor subtype 2 (SSTR-2) [15,16]. Transgenic mice with apolipoprotein E (ApoE) deficiency develop atherosclerotic plaques, validating the uptake of ^68^Ga-DOTATATE in plaques, as shown by autoradiography [17]. Furthermore, in histochemistry analysis, plaques showed high numbers of macrophages. These results could be reproduced by another atherosclerosis transgenic mouse model using low-density lipoprotein (LDL) receptor deficiency [18].

Moreover, cancer patients receiving ^64^Cu-DOTATATE and ^68^Ga-DOTATATE imaging showed relevant tracer uptake in large arteries and significant correlations to cardiovascular risk factors [19]. In the patient undergoing endarterectomy in the carotid artery, ^64^Cu-DOTATATE was exceptionally high on the symptomatic compared to the contralateral side [20]. However, conflicting data show the opposite observation in other symptomatic patients before the endarterectomy of the carotid artery [21]. Moreover, no SSTR-2 expression was evident in the explanted plaques in immunohistochemistry. Targeting vulnerable atherosclerotic plaques via a PET-tracer may enhance diagnostic imaging modalities [22]. Recently, a subgroup analysis of CT-guided plaque categories showed that more severe plaques correlate with tracer uptake [23]. Significant calcium scores were also associated with a higher uptake of ^68^Cu-DOTATATE in the left anterior descending artery [8]. Interestingly, no co-localization of ^68^Cu-DOTATATE and ^18^F-FDG has been published yet, hypothesizing that the tracers illustrate different processes in atherosclerosis. In summary, ^68^Cu-DOTATATE imaging illustrates distinct processes in atherosclerotic plaques and, therefore, hypothetically leads to novel diagnostic and therapeutic paths.

The peptide receptor radionuclide therapy (PRRT) is a validated tool in somatostatin receptor-positive cancer, e.g., neuroendocrine cancer. Therapy regimens use ^90^Yttrium (^90^Y) and ^177^Lutetium (^177^Lu) [24,25,26]. However, it remains unknown what effect the radiotracers have on other cells that express the SSTR-2 in calcified atherosclerotic plaques. In this study, we aimed to evaluate the effect of ^90^Y- and ^177^Lu-DOTATATE therapy on atherosclerotic plaque inflammation using serial ^68^Ga-DOTATATE PET/CT imaging and, therefore, adding data to the existing body of literature.

## 2. Materials and Methods

### 2.1. Patient Cohort

This retrospective study analyzed 57 oncologic patients having ^68^Ga-DOTATATE PET/CT imaging (carcinoid (*n* = 57), including pancreatic primary (*n* = 14), small intestine (*n* = 23), other primary (*n* = 11), unknown primary (*n* = 7), thymic carcinoma (*n* = 1), and thyroid cancer (*n* = 1). Overall, 37 patients received four PRRT cycles (^90^Y- or ^177^-Lu DOTATATE therapy), representing the therapy group. All patients had a baseline ^68^Ga-DOTATATE PET/CT before PRRT administration and a control PET/CT after 2 PRRT cycles. The other patients (*n* = 20) had regular staging scans using ^68^Ga-DOTATATE without PRRT (see Table 1). Patients receiving chemotherapy four weeks before PRRT with systemic rheumatic disease or vasculitis were excluded. In total, 171 PET/CTs were analyzed. This retrospective study was approved by the Ethikkommission der Medizinischen Fakultät der LMU München (protocol code 19-528 and date of approval 29 August 2019) and complied with the declaration of Helsinki.

### 2.2. ^68^Ga-DOTATATE Imaging Technique

Patients underwent ^68^Ga-DOTATATE PET/CT imaging in der Department of Nuclear Medicine, LMU Munich, on a Biograph TruePoint 64 (Siemens Health engineers, Erlangen, Germany). Prior to scanning, patients did not fast. ^68^Ga-DOTATATE was administered intravenously in a single bolus injection (appr. 200 MBq). Patients received 20 mg of furosemide to enhance diuresis. After 60 min of rest, the patients were transferred to the scanning suite for data acquisition. Transmission data were acquired via a low-dose CT scan (220 mAs, 120 kV, 512 × 512 matrix, 5 mm slice thickness, 5 mm/s increment, 0.5 s rotation time, 0.65 pitch index).

Three-dimensional (3D) PET emission scans were acquired with a 144 × 144 matrix. Following decay and scatter correction, PET data were reconstructed without attenuation correction.

### 2.3. Image Analysis

The imaging analysis was adapted, as described previously [27]. Eight distinct arterial regions were defined: ascending aorta, aortic arch, descending aorta, abdominal aorta, right iliac artery, left iliac artery, right carotid artery, and left carotid artery. Fused PET/CT images were used for the region of interest (ROI) analyses evaluating each region of the respective artery. A round ROI of 1 cm was used. Maximum standardized uptake values (SUV_max_) were measured, as described previously [27]. The mean of three blood-pool SUVs was calculated in the inferior vena cava and superior vena cava, respectively. The mean of the total six values was defined as the SUV_bloodpool_. The SUV_max_ was divided by the SUV_bloodpool_, yielding the target-to-background ratio (TBR) for each artery included in the analysis [9,28]. The overall vessel uptake (OVU) was defined as the sum of all TBR values. CT scans were evaluated for the calcified plaque in the same vessel wall of the same arterial segment investigated by ^68^Ga-DOTATATE PET. Calcification was semi-quantitatively ranked from 0 to 4, as described previously [29]. A score of 0 was assigned when the calcified plaque was absent; 1, defined as a small, calcified plaque covering less than 10% of the vessel circumference; 2, when the calcified plaque involved 10% to 25% of the vessel circumference; 3, when 25% to 50% of the vessel was calcified; and 4, when more than 50% of the vessel circumference was involved. CP scores are the sum of 8 respective areas, representing the ascending aorta, aortic arch, descending aorta, abdominal aorta, left and right iliac arteries, and left and right carotid arteries. The program Hybrid Viewer (Hermes Medical Solutions, Stockholm, Sweden) was used for image analysis.

### 2.4. Statistical Analysis

Statistics were performed using IBM SPSS Statistics (Version 21.0.0.0). The Shapiro–Wilk test evaluated normal distribution. The Student’s t-test analyzed data with normal distribution after the Levene test for variance analysis. A non-paired t-test was used to analyze the therapy and control groups. A paired t-test was used for the same group at different follow-up scans. A one-way ANOVA with post hoc Bonferroni was used to analyze more than two groups. Mann–Whitney U tests were used for nonparametric data. The Wilcoxon test was used to measure the differences between the two groups using repeated measurements. The Chi-Square test compared qualitative variables. Correlation analysis used Pearson’s r calculated as a measure of the linear correlation between two metric datasets, and Spearman for non-metric variables. *p* value < 0.05 was considered statistically significant.

## 3. Results

### 3.1. Patient Population

In total, the therapy and control groups consisted of 37 and 20 patients, respectively. The descriptive statistics comparing these groups did not evaluate significant differences, except the dose of the third PET/CT scan (control vs. therapy), and the time between the first and second scan (control vs. therapy) was different. The study cohort did not differ in other parameters, such as age, gender, diabetes mellitus, hypertension, and BMI. All patient parameters are listed in Table 2.

### 3.2. Correlation of Calcified Plaque Score, Overall Vessel Uptake (OVU), and Age

**Figure 1:**
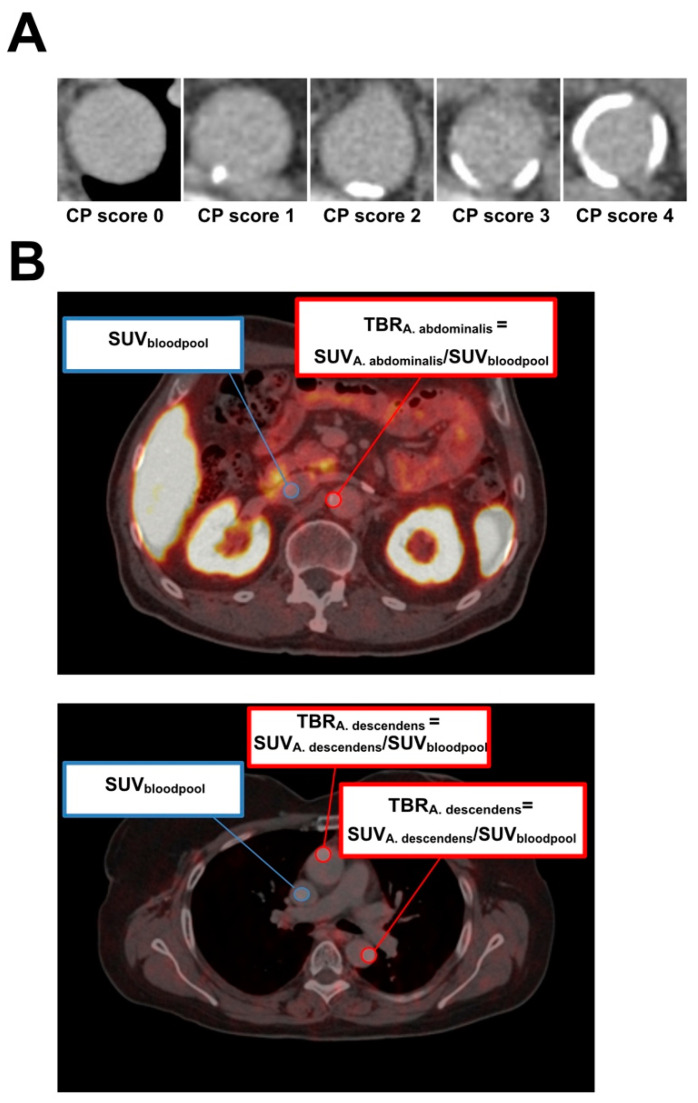
PET/CT image analysis. (**A**) Example of modified calcified plaque (CP) scoring system. Representative images show scores of 0 (no calcified plaque), 1 (calcified plaque involving 10% of vessel circumference), 2 (calcified plaque involving 10% to 25%), 3 (calcified plaque involving 25% to 50%), and 4 (calcified plaque involving >50%). (**B**) Representative image for target-to-background (TBR) calculation method. ROIs were drawn in the center of the vena cava inferior (blue) and arterial wall (red) for SUV evaluation.

First, the PET/CT images were evaluated for tracer uptake and CP score. Figure 1A illustrates the CP burden from scores 0 to 4. The SUV_bloodpool_, SUV_max_, and TBR were derived from the fused PET and CT images for the arteries included in the evaluation (e.g., ascending aorta, aortic arch, descending aorta, abdominal aorta, both iliac arteries, and both carotid arteries) (see Figure 1B).

**Figure 2:**
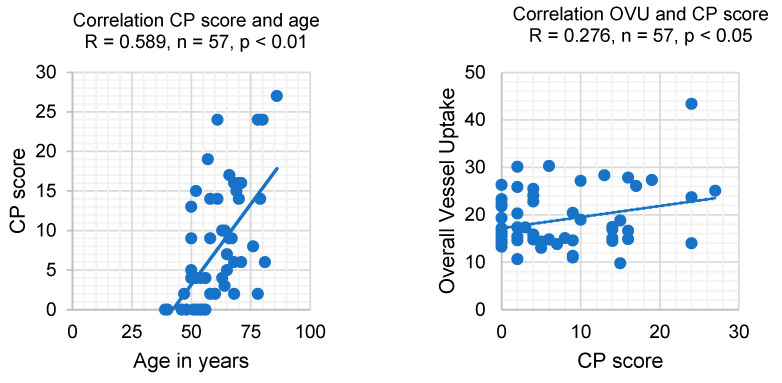
Correlation analysis of CP score, age, and OVU in the first PET/CT scan prior to PRRT therapy. *N* = 57.

Next, correlation analysis was performed for age, the OVU, and the CP scores at the first PET/CT scan prior to PRRT therapy. The sum of the therapy and control groups was used for analysis (*n* = 57). Our data show a positive correlation for the CP score to patient age (R = 0.589, *p* < 0.01) (Figure 2) and for the correlation of the CP score to the OVU (R = 0.276, *p* < 0.05) in the first PET/CT scan. Of note, there was no correlation between the OVU and age (R = 0.147, *p* = 0.275).

### 3.3. Correlation of Each Vessel Segment to OVU

Additionally, correlation studies for each vessel segment to the OVU were performed. This analysis should ensure the representability of the OVU in generalized atherosclerosis. Analysis of the TBR in all vessel segments to the OVU showed excellent R values (*p* < 0.001). (See Table 3). The best correlation was detected for the ascending aorta (R = 0.891; *p* < 0.001).

### 3.4. Longitudinal OVU Evaluation in the Therapy Group

**Figure 3:**
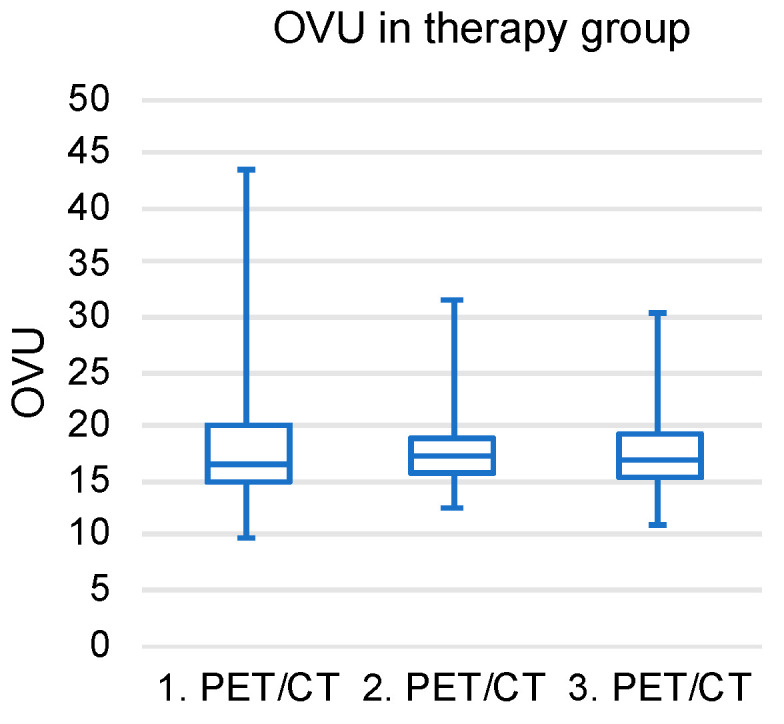
Boxplots illustrating the therapy group’s longitudinal assessment of the OVU, including the first to third ^68^Ga-DOTATATE scan.

In the next step, the OVU was analyzed in the therapy group to detect alterations in the ^68^Ga-DOTATAE uptake. The OVU analysis of the therapy group included 37 patients but did not show relevant changes in the follow-up scans (1. PET/CT vs. 2. PET/CT: *p* = 0.40; 2. PET/CT vs. 3. PET/CT: *p* = 0.51; 1. PET/CT vs. 3. PET/CT: *p* = 0.62; see Figure 3).

### 3.5. Tercile-Based Analysis of Patient Cohorts

In the next step, the therapy and control groups were ranked by their OVU value and separated into tercile-based subgroups, as described previously for the ^18^F-FDG assessment of atherosclerotic plaques [8]. Four subgroups were created based on the OVU: therapy group low (TG_low), therapy group high (TG_high), control group low (CG_low), and control group high (CG_high). The subgroups were compared by descriptive statistical analysis (see Table 4). The baseline characteristics were equally distributed. No significant differences were found among the tercile-based subgroups. The middle tercile was not used for further analysis.

**Figure 4:**
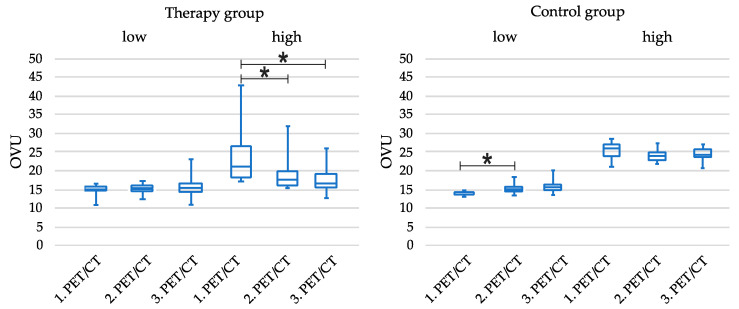
Boxplot illustration of the OVU course in therapy and control group in all three PET/CTs. * *p* < 0.05.

The tercile-based subgroups intra-group analysis showed no change in the TG_low group from scan one to three but, interestingly, a significant decrease in the TG_high group (1. PET/CT vs. 2. PET/CT; *p* = 0.04 and 1. PET/CT vs. 3. PET/CT; *p* = 0.03). Furthermore, in the CG_low group, the OVU changed (1. PET/CT vs. 2. PET/CT; *p* = 0.04; Table 5 and Figure 4). In inter-group analysis, there was no difference comparing the TG_high and CG_high groups at the 1. PET/CT baseline scan. The OVU was significantly lower in the 2. and 3. PET/CT scan in the TG_high group compared to the CG_high group (each *p* < 0.001). In addition, there was no statistical difference when comparing the TG_low and CG_low groups.

## 4. Discussion

Detrimental cardio- and cerebrovascular events, e.g., myocardial infarction or cerebral stroke, mainly result from ruptured atherosclerotic plaques. Without imaging techniques, it is impossible to identify the plaques prone to rupturing, the so-called vulnerable plaques [30,31]. Inflammation in atherosclerotic plaques plays an immense role in this context (reviewed in [32]). Combining PET and CT, or MR for hybrid imaging, enables the evaluation of not only morphological but more critical biological functional data. DOTATATE represents a nuclear tracer binding to the somatostatin receptor, which is also present in immune cells, especially in macrophages [16]. Macrophages play a pivotal role in atherosclerosis [33]. This retrospective study wanted to assess the feasibility of DOTATATE imaging for atherosclerotic plaques and evaluate the impact of PRRT therapy on atherosclerotic plaques. This work shows a decline in tracer uptake in the therapy subgroup with initially high values. At baseline, the OVU moderately correlates with the calcified plaque score, thus enabling a diagnostic approach. However, it should be mentioned that this study did not classify if the atherosclerotic plaques showed, e.g., a high immune cell count in histology. This could partially explain the moderate statistic for correlation analysis by potentially summating low- and high-inflammatory plaques.

Previous retrospective and prospective clinical studies could show the feasibility of PET/CT for atherosclerotic imaging using ^18^F-FDG [7,34,35,36]. Studies using ^18^F-FDG managed to show the degree of inflammation in atherosclerotic plaques [13]. However, there is also a specific range in variability derived from patient preparation, blood glucose levels, the injected dose, and the time between the ^18^F-FDG injection and data acquisition [37]. Besides these difficulties, patients are not allowed to eat six hours before the scans, and some patients suffer from diabetes. However, diabetic patients are prone to develop atherosclerosis. Another limitation is the ^18^F-FDG uptake in the heart, further limiting the assessment of the coronary arteries (reviewed in [38]).

Several previous studies show the feasibility of ^18^F-NaF for imaging coronary atherosclerosis (reviewed in [38]). Consequently, other tracers were evaluated for feasibility. In patients with cancer, a retrospective evaluation showed ^18^F-NaF uptake into the vascular system imaging atherosclerotic plaques [39] and association with cardiovascular risk factors and the Framingham risk score [40,41,42]. Compared to the ^18^F-FDG uptake, there was a higher uptake of ^18^F-NaF in the vessels [43]. Also, in prospective studies, ^18^F-NaF uptake was significantly higher in patients with known coronary artery disease (CAD) compared to ^18^F-FDG [44]. Interestingly, in patients with recent myocardial infarction, a distinct ^18^F-NaF uptake in the area of the vulnerable plaque could be shown [45]. Another study could show a higher target-to-background ratio in vessels with vulnerable plaques than in small vessels [46].

The cellular target in macrophages, the somatostatin receptor subtype 2, could be a promising approach [15,16]. Thus, imaging the inflammatory activity in atherosclerotic plaques could be conducted without the limitation of myocardial uptake, compared to ^18^F-FDG [12,36]. The first prospective study (VISION study: vascular inflammation using somatostatin receptor positron emission tomography) in non-oncologic patients suffering from coronary heart disease and acute coronary syndrome aimed to investigate this question.

^68^Ga-DOTATATE could more reliably identify vulnerable plaques compared to ^18^F-FDG [36]. Tarkin et al. could show that ^68^Ga-DOTATATE represents the activity of macrophages [36]. Major adverse events, such as myocardial infarction and stroke, result from plaque rupture in coronary and carotid arteries, respectively. The detection of vulnerable coronary and carotid plaques, as previously described by Tarkin et al., may tremendously improve risk assessment and clinical follow-up in patients with cardiovascular disease. Therefore, it remains the question if PRRT therapy displayed by ^90^Y-DOTATATE and ^177^Lu-DOTATATE can also target macrophages and lead to a modulation of inflammatory processes in atherosclerotic plaques. Schatka et al. recently detected an OVU decrease after PRRT therapy in a small cohort of 11 patients [47]. Different tracer uptake times (30 ± 10 min) and ^68^Ga-DOTATATE activities (73 ± 13 MBq) make a direct comparison of the studies challenging. The higher OVU in Schatka et al. could be due to patients with high cardiovascular risk. Nevertheless, both studies could show a decrease in the OVU after PRRT, especially in patients with high OVU values. This OVU drop underlines the possibility of new therapeutic approaches, such as immune cell modulation by target-specific tracers.

### Limitations

The high-sensitive (hs) C-reactive protein (hs-CRP) as a biomarker for atherosclerosis was not evaluated in this study. Due to the nature of the retrospective study, not all the cardiovascular risk factors and events and atherosclerotic sequels could potentially be derived from the records. In addition, our study population, which consists of oncologic patients, is not suitable for creating a reliable hypothesis regarding hsCRP change. Another limitation is the moderate number of patients included in the study (*n* = 57). Another limitation is the lack of standard to assess inflammation in vessels and as well the lack of assessment of the plaque morphology beyond that of calcification, since contrast was not given during CT acquisition, which may further enable more accurate plaque assessment.

## 5. Conclusions

This retrospective study assessed the effect of ^90^Y-DOTATATE and ^177^Lu-DOTATATE on atherosclerotic plaque remodeling in oncologic patients by monitoring ^68^GA-DOTATATE uptake in serial longitudinal PET/CT follow-up scans. We could detect a decline in ^68^GA-DOTATATE activity in the tercile therapy cohort with an initially high tracer uptake. The OVU shows a solid correlation to the TBR in all arterial segments. The best correlation of the TBR in the ascending aorta could serve as the reference value for atherosclerotic plaque inflammation. Our results support the notion that PRRT could modulate atherosclerotic plaque inflammation and might, therefore, be an interesting therapeutic approach in the future.

## Figures and Tables

**Table 1:** Study design of the retrospective evaluation.

Study Design	
Therapy Group	Control Group
1. ^68^Ga-DOTATATE PET/CT (Baseline)	1. ^68^Ga-DOTATATE PET/CT (Baseline)
1. Cycle PRRT	
2. Cycle PRRT	
2. ^68^Ga-DOTATATE PET/CT	2. ^68^Ga-DOTATATE PET/CT
3. Cycle PRRT	
4. Cycle PRRT	
3. ^68^Ga-DOTATATE PET/CT	3. ^68^Ga-DOTATATE PET/CT

**Table 2:** Descriptive statistics illustrating baseline characteristics of therapy and control group. Nominal variables are displayed as numbers/percentages, and metric variables are shown as mean values ± standard deviation. Years (y), days (d), megabecquerel (MBq).

Descriptive Statistics for Therapy and Control Group			
	Therapy Group (*n* = 37)	Control Group (*n* = 20)	*p*-Value
Age [y]	59 ± 10	63 ± 12	0.17
Male	23 (62.2%)	9 (45%)	0.268
Diabetes mellitus	6 (16.2%)	4 (20%)	0.728
Hypertension	26 (70.3%)	12 (60%)	0.558
BMI [kg/m^2^]	25.9 ± 4.8	27.5 ± 4.9	0.198
OVU	18.5 ± 6.7	19.6 ± 5.7	0.707
CP score	6 ± 6	10 ± 9	0.156
Dose 1. PET/CT [MBq]	206 ± 27	208 ± 31	0.841
Dose 2. PET/CT [MBq]	218 ± 20	221 ± 29	0.735
Dose 3. PET/CT [MBq]	211 ± 23	229 ± 40	0.036
Cycle count ^177^Lutetium	136		
Dose ^177^Lutetium [MBq]	7254 ± 640		
Cycle count ^90^Yttrium	12		
Dose ^90^Yttrium [MBq]	3647 ± 189		
Time between 1st and 2nd PET/CT [d]	214 ± 68	174 ± 76	0.019
Time between 2nd and 3rd PET/CT [d]	407 ± 356	238 ± 100	0.408
Time between 1st and 3rd PET/CT [d]	621 ± 401	413 ± 114	0.126

**Table 3:** Correlation analysis of TBR and OVU for each vessel segment.

Correlation Analysis of Each TBR to OVU	
Vessel segment	
TBR ascending aorta	R = 0.891; *p* < 0.001
TBR aortic arch	R = 0.825; *p* < 0.001
TBR descending aorta	R = 0.887; *p* < 0.001
TBR abdominal aorta	R = 0.877; *p* < 0.001
TBR right iliac arteries	R = 0.853; *p* < 0.001
TBR left iliac arteries	R = 0.804; *p* < 0.001
TBR right carotid artery	R = 0.784; *p* < 0.001
TBR left carotid artery	R = 0.848; *p* < 0.001

**Table 4:** Descriptive statistics of several subgroups based on tercile split. Metric variables are displayed as mean ± standard deviation. Nominal variables are illustrated as the number/percentage of the whole group. Years (y), days (d), megabecquerel (MBq).

**Descriptive Statistics for Subgroups**					
	**Therapy Group (TG)**		**Control Group (CG)**		***p* Value**
Tercile	Low	High	Low	High	
Count	12	12	10	10	
Age [y]	57 ± 12	62 ± 10	63 ± 12	64 ± 12	0.577
Men	6 (50%)	9 (75%)	3 (30%)	6 (60%)	0.197
Diabetes mellitus	1 (8.3%)	2 (16.7%)	3 (30%)	1 (10%)	0.519
Hypertension	5 (41.7%)	11 (91.7%)	6 (60%)	6 (60%)	0.082
BMI [kg/m^2^]	24.2 ± 4.7	25.9 ± 3.8	26.7 ± 5.9	28.4 ± 3.8	0.221
OVU	14.8 ± 1.4	23.7 ± 7.7	14.4 ± 0.6	24.9 ± 2.6	<0.001
CP score	4.6 ± 5.2	7.2 ± 7.2	7.2 ± 7.5	12.1 ± 9.7	0.146
Dose 1. PET/CT [MBq]	205 ± 30	200 ± 31	205 ± 36	210 ± 26	0.897
Dose 2. PET/CT [MBq]	219 ± 19	225 ± 20	217 ± 31	225 ± 28	0.821
Dose 3. PET/CT [MBq]	207 ± 20	214 ± 17	232 ± 30	227 ± 50	0.226
Time from 1. to 3. PET/CT [d]	438 ± 94	370 ± 70	412 ± 86	413 ± 142	0.425

**Table 5:** OVU course from first to third PET/CT in the different subgroups (therapy and control group) after tercile splits. Data represent mean ± standard deviation.

OVU Course			
	1. PET/CT	2. PET/CT	3. PET/CT
TG_low	14.76 ± 1.44	15.4 ± 1.66	15.98 ± 3.11
TG_high	23.69 ± 7.72	18.88 ± 4.64	18.7 ± 4
CG_low	14.42 ± 0.56	15.45 ± 1.47	16.22 ± 2.03
CG_high	24.89 ± 2.56	24.12 ± 1.93	24.09 ± 1.93

## Data Availability

Further information and requests for resources and data should be directed to and will be fulfilled by the lead contact Maximilian Fischer (maximilian.fischer@med.uni-muenchen.de).
